# The CD300c antibody CL7 suppresses tumor growth by regulating the tumor microenvironment in non-small cell lung carcinoma

**DOI:** 10.3389/fonc.2025.1698857

**Published:** 2025-11-25

**Authors:** Soyoung Kim, Ik-Hwan Han, Suin Lee, Sujin Park, Jae-Won Jeon, Hyunsu Bae

**Affiliations:** 1 Department of Physiology, College of Korean Medicine, Kyung Hee University, Seoul, Republic of Korea; 2 CentricsBio Inc., Seoul, Republic of Korea

**Keywords:** non-small cell lung carcinoma, CD300c, CL7, tumor-associated macrophage, tumor microenvironment, immunotherapy

## Abstract

Despite advances in therapy, non-small cell lung cancer (NSCLC) continues to rank among the deadliest cancers worldwide. Targeting immunosuppressive components within the tumor microenvironment (TME) has emerged as a promising therapeutic strategy. Unlike M1 tumor-associated macrophages (TAMs), M2-like TAMs contribute to NSCLC progression by promoting an immunosuppressive tumor microenvironment (TME), highlighting the need for tumor microenvironment remodeling. CL7, a monoclonal antibody that targets the activating receptor CD300c on human monocytes and macrophages, was selected as a therapeutic candidate because CD300c engagement triggers MAPK and NF-κB signaling pathways, promoting M1 macrophage polarization and antitumor immune activation. To evaluate the therapeutic potential of CL7, we established an orthotopic NSCLC model by inoculating LLC-luc cells into the left lung of mice. We administered CL7 intraperitoneally at doses of 5 or 10 mg/kg twice a week. Only representative data from the 10 mg/kg CL7 group are shown to maintain consistency with subsequent analyses (flow cytometry, RT-qPCR, and IHC). Tumor growth was significantly suppressed in the CL7-treated group compared to the PBS control group. CL7 treatment also modulated the tumor microenvironment by increasing the population of M1 macrophages and CD8^+^ T cells, while decreasing the population of regulatory T cells. Our findings suggest that CL7 exerts antitumor effects in NSCLC by reprogramming the immunosuppressive landscape of the TME and enhancing antitumor immunity.

## 1 Introduction

Lung cancer is one of the most lethal cancers for both men and women. Regardless of clinical stage, the overall prognosis of lung cancer remains poor, with a 5-year survival rate of approximately 22% (1). Lung cancer is broadly classified into two main subtypes: small cell lung carcinoma (SCLC) and non-small cell lung carcinoma (NSCLC), accounting for approximately 15% and 85% of all cases, respectively (2).

The tumor microenvironment (TME) is composed of tumor cells, stroma, immune cells, blood vessels, and extracellular matrices (3, 4). The TME plays a pivotal role in tumor initiation and progression of NSCLC by regulating key processes such as tumor cell growth, immune evasion, and angiogenesis (5–7). Cancer immunotherapy aims to enhance the immune system to target and eliminate tumor cells, while also modifying the TME to improve treatment outcomes (8). Immune checkpoint inhibitors targeting PD-1, PD-L1, and CTLA-4 have transformed NSCLC treatment; however, many patients remain unresponsive or develop acquired resistance. Immunotherapy has demonstrated long-term efficacy and tolerability across several cancers, making it a promising therapeutic approach. However, immunotherapy is often limited in efficacy, with immune escape and drug resistance emerging as challenges, particularly due to factors inherent within the TME (7, 9). Therefore, exploring novel immunotherapeutic strategies is essential to overcome these limitations and enhance treatment outcomes.

Tumor-associated macrophages (TAMs) are a major component of NSCLC TME. Most TAMs originate from monocytes circulating in the bloodstream and exhibit significant plasticity, with their phenotypes shaped by signals within the TME (10–13). TAMs can be divided into classically activated M1 macrophages and alternatively activated M2 macrophages. High-density infiltration of M2 TAMs in the tumor stroma has been associated with tumor growth, angiogenesis, invasion, metastasis, and poor prognosis in lung cancer, whereas M1 macrophages are typically located in tumor islets and related to good prognosis (14, 15). Given their pivotal role in tumor progression, TAMs have become key targets for therapeutic strategies. Several therapeutic strategies have been developed to manipulate TAMs in cancer, including 1) TAM recruitment inhibition in the TME, 2) shifting their phenotype from tumor-promoting M2 to tumor-suppressive M1, 3) selective elimination of M2 TAMs, and 4) harnessing TAMs for drug delivery (16–18).

CD300 proteins are expressed on a variety of immune cells, where they are involved in both stimulatory and inhibitory signaling functions (19). CD300c, a member of the CD300 family, is expressed on the surface of human monocytes and monocyte-derived cells, including macrophages and dendritic cells. Recent studies have highlighted the dual immunomodulatory roles of CD300 family members in tumor immunity, with CD300a acting as an inhibitory checkpoint and CD300c serving as an activating receptor that promotes pro-inflammatory responses in myeloid cells (20). Previous studies have demonstrated that targeting CD300c with a monoclonal antibody activates MAPK and NF-κB pathways, promoting M1 macrophage polarization and suppressing tumor growth in preclinical models (21). Given the involvement of CD300c in macrophage polarization and antitumor responses, targeting this receptor may offer a novel strategy to modulate the NSCLC microenvironment.

In this study, we aimed to evaluate whether CL7 (CB301) treatment could suppress tumor growth and progression in NSCLC by modulating immune cell composition within the tumor microenvironment.

## 2 Materials and methods

### 2.1 Reagents and cell lines

This biopanning process was performed to obtain a CD300c-specific antibody clone with high affinity and binding specificity. The clone CL7 was isolated from four rounds of biopanning against human CD300c using synthetic human scFv library based on VH3–23 and VL1-47, with non-combinant complementarity determining region (CDR) diversity (unpublished results). Lewis-lung carcinoma (LLC) cells were purchased from ATCC. LLC cells were maintained in DMEM supplemented with 1% penicillin-streptomycin (Hyclone) and 10% fetal bovine serum (FBS; Welgene). Cells were subcultured every 2–3 days to ensure proper growth of cells. All cells were cultured in a humidified incubator set at 37°C. Luciferase-expressing LLC cells (LLC-luc) were generated by transducing parental LLC cells with a lentiviral vector encoding the firefly luciferase gene (luc2), enabling bioluminescence imaging for *in vivo* tumor monitoring.

### 2.2 Animal study

Male wild-type C57BL/6J (6–8 weeks) mice were purchased from DBL (Chungbuk, Republic of Korea). All animal experiments were approved by the Institutional Animal Care and Use Committee (IACUC) of Kyung Hee University (Approval number: KHUASP(SE)-24-041), and were conducted in accordance with the ARRIVE guidelines and the NIH Guide for the Care and Use of Laboratory Animals (NIH Publications No. 8023, revised 1978). Male mice were used in this study to ensure higher surgical tolerance and survival rates during orthotopic tumor implantation procedures. Additionally, male mice exhibit more consistent body weights and reduced hormonal variability compared to females, which can minimize experimental variability in tumor growth assessment. All animals were maintained in a pathogen-free environment with a 12 h light/dark cycle and were supplied with water and food ad libitum.

To generate the mouse lung cancer orthotopic model, a 30 µL suspension of 2 × 10^4^ LLC-Luc cells in Matrigel matrix (Corning, NY, USA) were prepared. Cells were suspended in serum-free DMEM and mixed with Matrigel. Matrigel: cell suspension at a 6:4 (vol/vol) ratio. Mice were anesthetized with 3% isoflurane in an induction chamber, then placed on a sterile heating pad. Then positioned in a left lateral decubitus position with his nose in an isoflurane (3%) nosecone to maintain anesthesia during surgery. The left dorsal side of the mouse, which was removed of all hair the previous day using Nair, was prepared for sterile surgery by wiping with ethanol and then betadine three times. A small incision was made horizontally using scissors to cut through the skin, and to carefully cut through the underlying fat layer. A 30 µL suspension of 2 × 10^4^ LLC-Luc cells were injected into the injection site of the left lung (between the fifth and sixth rib bones) using insulin syringe. To prevent the hemorrhage and the cellular spill after injection, the transient local compression was operated. After that, the skin was closed back together using 3M Vetbond Tissue Adhesive. The dosing schedule and concentrations for CL7 administration were determined based on our previous preclinical study using a CD300c-targeting antibody (21). Three days after inoculation, mice were intraperitoneally injected with CL7 (5 and 10 mg/kg (CentricsBio)) while measuring the resulting tumor size changes. For measurement, D-luciferin (150 mg/kg) was injected intraperitoneally into the mice, and imaging was performed 10 minutes later using an *in vivo* imaging system (Berthold Technologies). After four drug injections, tumor growth was assessed, and mice were deeply anesthetized with isoflurane and euthanized by cervical dislocation (n=9). For all analyses, total lung tissues containing tumor lesions were harvested and used for flow cytometry and molecular assays.

To generate the mouse lung cancer subcutaneous model, a 100 µL suspension of 1 × 10^5^ LLC cells in Matrigel matrix (Corning, NY, USA) were prepared and inoculated subcutaneously into the right flank. When the tumor volume reaches the 50mm^3^, mice were intravenously injected CL7 (10mg/kg ((CentricsBio)) every three days (n=7). Mice were sacrificed when the tumor size attained a maximum diameter of 1.5 cm after inoculation.

### 2.3 Measurement of tumor growth in NSCLC orthotopic model

In the LLC orthotopic model, the tumor growth was detected using an *in vivo* bioluminescence imaging system (NightOWL II; Berthold Technologies GmbH, Wildbad, Germany), at the end of the drug administration. Fifteen minutes after D-luciferin (BioVision, Milpitas, CA, USA) injection (4 mg per mouse), luminescence signals were detected with an exposure time of 0.1 s and 4 × 4 binning. Photon energy and tumor area were analyzed using IndiGO software (Berthold Technologies GmbH).

### 2.4 Flow cytometry

For flow cytometry analysis, lung tissues were harvested from the sacrificed mice and put in the MACS C tube (Miltenyi Biotec, Auburn, CA, USA) containing Collagenase D (1 mg/mL; Sigma-Aldrich, St. Louis, MO, USA) and DNase1 (1 mg/mL; Sigma-Aldrich) in serum-free medium. The tissues were dissociated using a MACS dissociator (Miltenyi Biotec) and digested for 25 min at 37°C with a shaking incubator. The tissues were then filtered using a 40 μm cell strainer (Corning Incorporated, Corning, NY, USA) to obtain a single-cell suspension. Red blood cells (RBCs) were lysed with 1× RBC lysis buffer (Invitrogen, Carlsbad, CA, USA) for 5 min at room temperature. Cells were washed and resuspended in BD Pharmingen™ Stain Buffer (BD bioscience, San Jose, CA, USA). The cells were stained for 45 min at 4 °C with antibodies. Gating boundaries were defined based on unstained and single-stained controls used for compensation and negative reference. The following antibodies were purchased from BD Biosciences or Biolegend (San Diego, CA, USA) for the identification of myeloid cells: mouse CD45-FITC, CD11b-PECy7, CD86-BV786, CD206-APC, F4/80-PE, and CD11c-APC-Cy7; to identify the infiltration of lymphoid cells: mouse CD45-APC-Cy7, CD8-PE-Cy7, CD4-BB700, CD25-BV421, and Granzyme B-APC. For intracellular staining, the cells were treated with 1x fixation and permeabilization buffer (BD Biosciences) for 30 min. The single-cell suspension was washed and stained with Granzyme B. The data were acquired using a BD FACSlyric™ (BD Biosciences) flow cytometry system and analyzed using the BD FACSuite software (BD Biosciences).

### 2.5 Quantitative real-time PCR

Total RNA was isolated from lung tissues of tumor-bearing mice using an easy-BLUE RNA extraction kit (iNtRON Biotechnology, Seongnam, South Korea). cDNA was synthesized using Cyclescript reverse transcriptase (Bioneer, Daejeon, South Korea) according to the manufacturer’s instructions. Real-time PCR was performed using a CFX connect real-time PCR system (Bio-Rad La-boratories, Hercules, CA, USA) and the SensiFAST SYBR no-Rox kit (Bioline, London, UK). The expression levels of the target mRNAs were normalized to the expression levels of mouse GAPDH, a housekeeping gene. All fold-changes were expressed relative to the PBS group. Each reaction was performed in duplicates. The base sequences of the primers used were listed in **Supplementary Table 1**.

### 2.6 Immunohistochemistry

Lung tissues from tumor-bearing mice were fixed in 10% neutral-buffered formalin, embedded in paraffin, and sectioned at a thickness of 5 μm. Deparaffinization was performed using xylene for 10 minutes, followed by rehydration through a graded ethanol series. Antigen retrieval was carried out by heating the sections in 10 mM sodium citrate buffer (pH 6.0) using a microwave. After washing with PBS, the sections were blocked with normal serum provided in the VECTASTAIN^®^ ABC-HRP Kit (PK-6101, Vector Laboratories, Newark, CA, USA) for 30 minutes. The sections were then incubated overnight at 4°C with a rabbit polyclonal anti-PCNA antibody (1:200, Santa Cruz Biotechnology, Dallas, TX, USA). Following PBS washes, a biotinylated secondary antibody was applied, and the sections were incubated with the VECTASTAIN^®^ ABC reagent. The signal was visualized using DAB peroxidase substrate kit (SK-4100, Vector Laboratories). Slides were rinsed in tap water, counterstained with hematoxylin, dehydrated, and mounted with coverslips. Tissue slides were photographed at 20× magnification under a light microscope (Olympus, Tokyo, Japan). Three random fields were selected, and PCNA-positive cells were quantified using QuPath software.

### 2.7 Statistics

The data collected were analyzed using Prism 5.01 software (GraphPad Software Inc., San Diego, CA, USA), and are expressed as the mean ± standard error of mean (SEM). All data were tested for normality using the normality test in GraphPad prism. Unpaired Student’s t-test was used to determine significant differences between two groups. Kaplan-Meier survival analysis was performed in the mouse NSCLC model (n=7). The statistical significance of the survival between mice treated with PBS and CL7 was determined using the log-rank (Mantel-Cox) test. Two-way ANOVA followed by Bonferroni *post-hoc* test was performed for group comparisons (n=9). P < 0.05 was considered to indicate a statistically significant difference.

## 3 Results

### 3.1 CL7 treatment significantly inhibits tumor growth and progression in an NSCLC model

**Figure 1:**
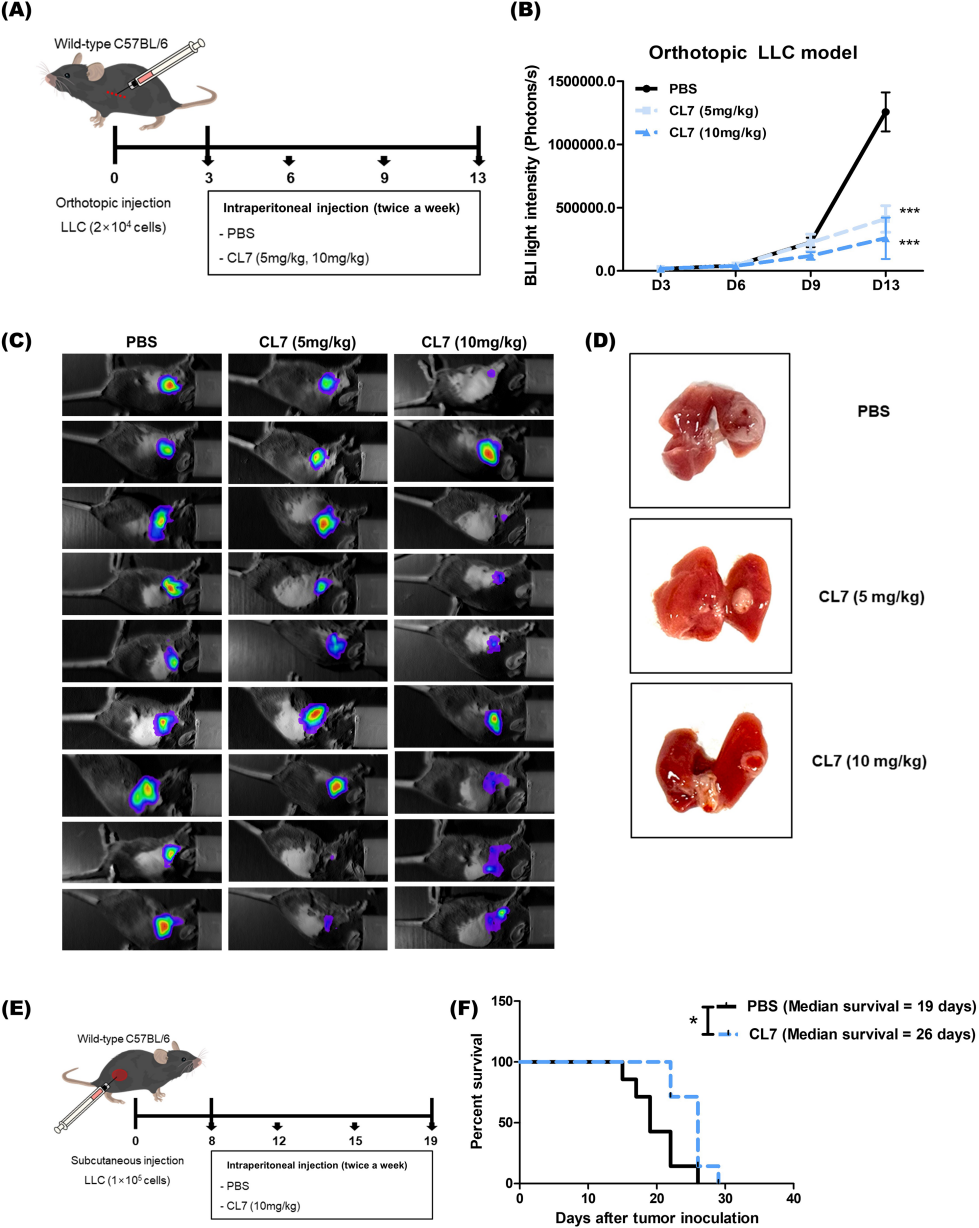
CL7 treatment significantly inhibits tumor growth and progression in an NSCLC model. **(A)** Schematic diagram of the *in vivo* experimental schedule. LLC-luc cells (2 × 10^4^) were injected into the left lung (between the fifth and sixth ribs) using an insulin syringe. Three days post-inoculation, mice received intraperitoneal injections of CL7 (5 or 10 mg/kg; CentricsBio) twice a week. The PBS was administered on the same schedule as the CL7 treatment. **(B, C)** Tumor progression was monitored using *in vivo* bioluminescence imaging after four drug administrations. **(D)** Mice were sacrificed, and lung tissues were collected for analysis. Only representative data from the 10 mg/kg CL7 group are shown to maintain consistency with subsequent analyses. All data are presented as mean ± SEM. *p < 0.05, ***p < 0.001 versus PBS group (n = 9). **(E)** In a subcutaneous model, regular LLC cells (1 × 10^5^) were injected into the right flank of mice. When tumor volumes reached 50 mm³, mice were intravenously treated with CL7 (10 mg/kg) every three days. Mice were sacrificed once tumor diameters reached 1.5 cm. **(F)** Kaplan-Meier survival analysis was performed in the orthotopic NSCLC model. Median survival was 19 days for the PBS group and 26 days for the CL7-treated group (n = 7). *p < 0.05 versus PBS group (log-rank test).

To investigate the effect of CL7 treatment on tumor growth in a non-small cell lung cancer (NSCLC) model, we established an orthotopic NSCLC mouse model and administered CL7 at doses of 5 or 10 mg/kg (**Figure 1A**). Both CL7-treated groups exhibited a significant reduction in tumor size compared to the PBS-treated control group. Although tumor size tended to be smaller in the 10 mg/kg group than in the 5 mg/kg group, the difference between the two treatment groups was not statistically significant (**Figures 1B-D**). Furthermore, mice treated with CL7 (10 mg/kg) showed a significant increase in survival compared to PBS-treated controls, with median survival was 19 and 26 days, respectively (**Figures 1E, F**). Our findings suggest that CL7 treatment reduces tumor growth and prolongs survival in the NSCLC model.

### 3.2 CL7 reduces NSCLC cancer cell proliferation

**Figure 2:**
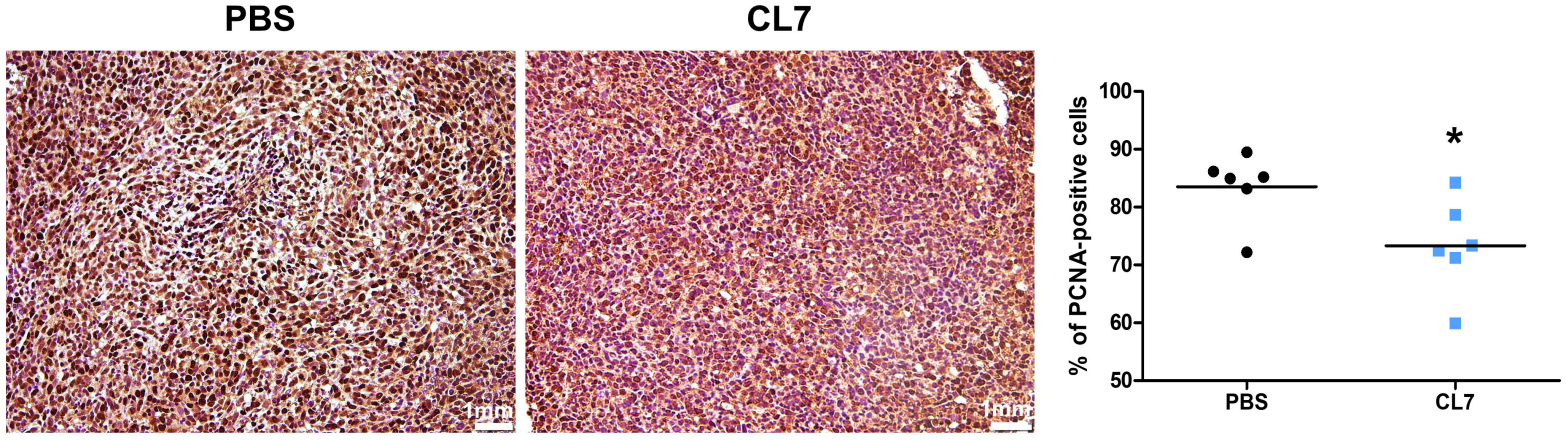
CL7 reduces NSCLC cancer cell proliferation. IHC images and quantitative analyses were performed using the 10 mg/kg CL7 group, which served as the representative dose for downstream experiments. Immunohistochemistry analysis for proliferating cell nuclear antigen (PCNA), a marker of cell proliferation, was performed on lung tissue sections from tumor-bearing mice (n = 6 per group). Images were acquired using a light microscope at 20× magnification. Three random fields were selected, and PCNA-positive cells were quantified using QuPath software. All data are presented as mean ± SEM. *p < 0.05 versus PBS group.

To investigate whether CL7 affects the tumor cell proliferation in NSCLC, the expression of PCNA, a proliferation marker in tumor tissues were examined by immunohistochemistry. The PCNA expression was reduced in a CL7-treated group compared to PBS group. In line with the reduced tumor growth, PCNA-positive cells were significantly decreased in CL7 group (**Figure 2**). These results demonstrate that CL7 inhibit the tumor growth and proliferation.

### 3.3 CL7 promotes M1 macrophage polarization within the NSCLC tumor microenvironment

**Figure 3:**
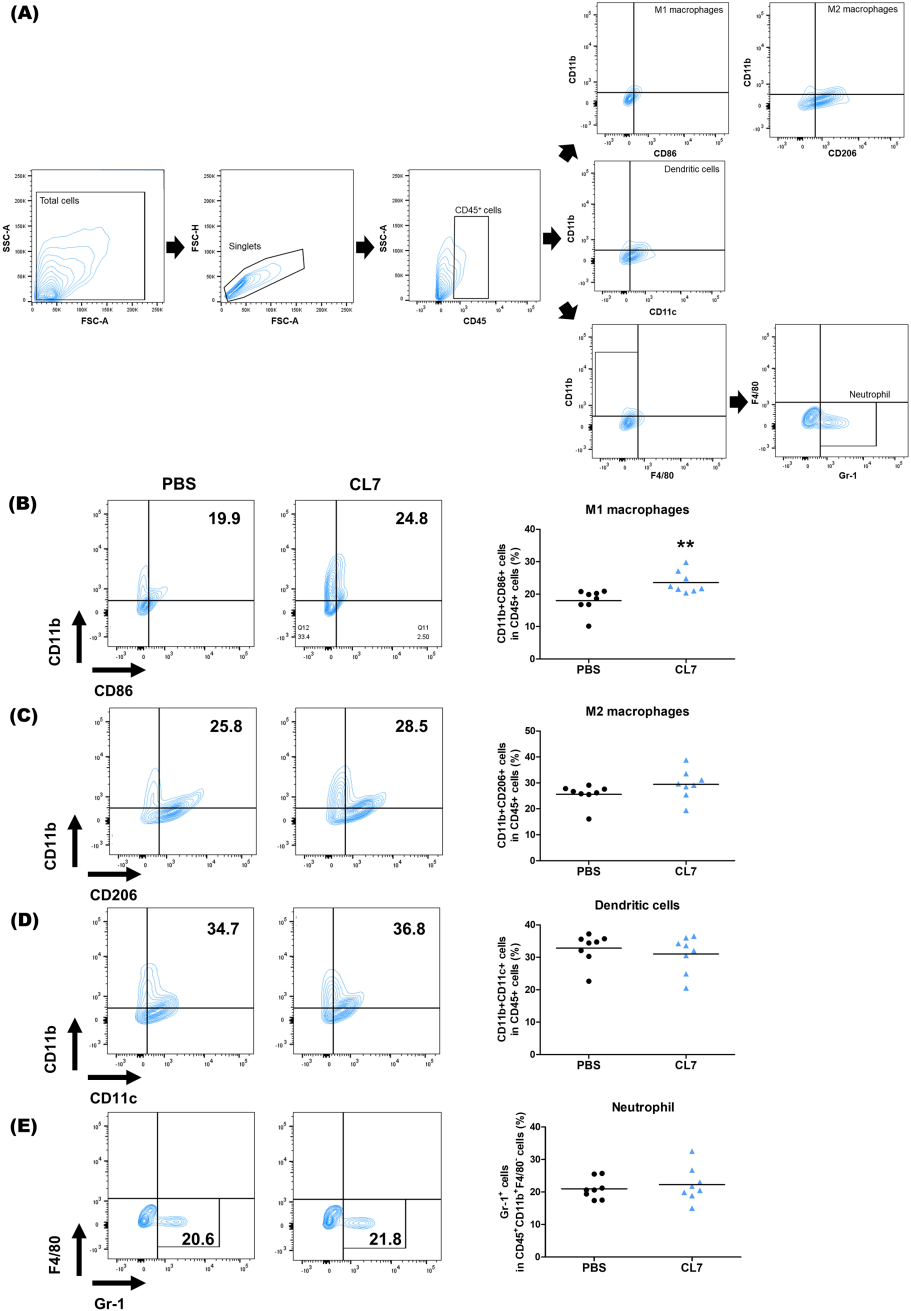
CL7 promotes M1 macrophage polarization within the NSCLC tumor microenvironment (TME). Identification of changes in immune cell populations within the NSCLC orthogonal mouse model by flow cytometry. Flow cytometry data were obtained from the 10 mg/kg CL7 group, which was used as the representative dose for downstream analyses. Gating boundaries were defined based on unstained and single-stained controls used for compensation and negative reference. **(A)** Identification of myeloid cell populations following exclusion of doublets. CD45^+^ gate was also used as a first step for specific immune cell identification. **(B)** CD45^+^CD11b^+^CD86^+^ cells were regarded as M1 macrophages, whereas **(C)** CD45^+^CD11b^+^CD206^+^ cells were regarded as M2 macrophages. **(C)** CD45^+^CD11b^+^CD11c^+^ cells were considered as dendritic cells (DCs) and **(D)** CD45^+^CD11b^+^F4/80^-^Gr-1^+^ were considered as neutrophils (n = 8 per group). All data are presented as the mean ± SEM; ** *p* < 0.01 versus PBS group.

CL7, the CD300c monoclonal antibody induced the repolarized M1 macrophages from monocytes (21). To determine whether CL7 influences in the modulation of macrophage population within the NSCLC tumor microenvironment (TME), we analyzed the macrophage population using flow cytometry in lung tissues of tumor-bearing mice (**Figure 3A**). The CL7 treatment group showed a significant increase in the M1 macrophage population compared to PBS group (**Figure 3B**), while there were no significant differences in the M2 macrophages (**Figure 3C**), dendritic cells (DCs) (**Figure 3D**), and neutrophils (**Figure 3E**). These results indicate that CL7 treatment promotes an M1-like macrophage phenotype within the tumor microenvironment, with minimal effects on dendritic cells, neutrophils, or M2 macrophages.

### 3.4 CL7 alleviates immunosuppressive conditions in the NSCLC TME

**Figure 4:**
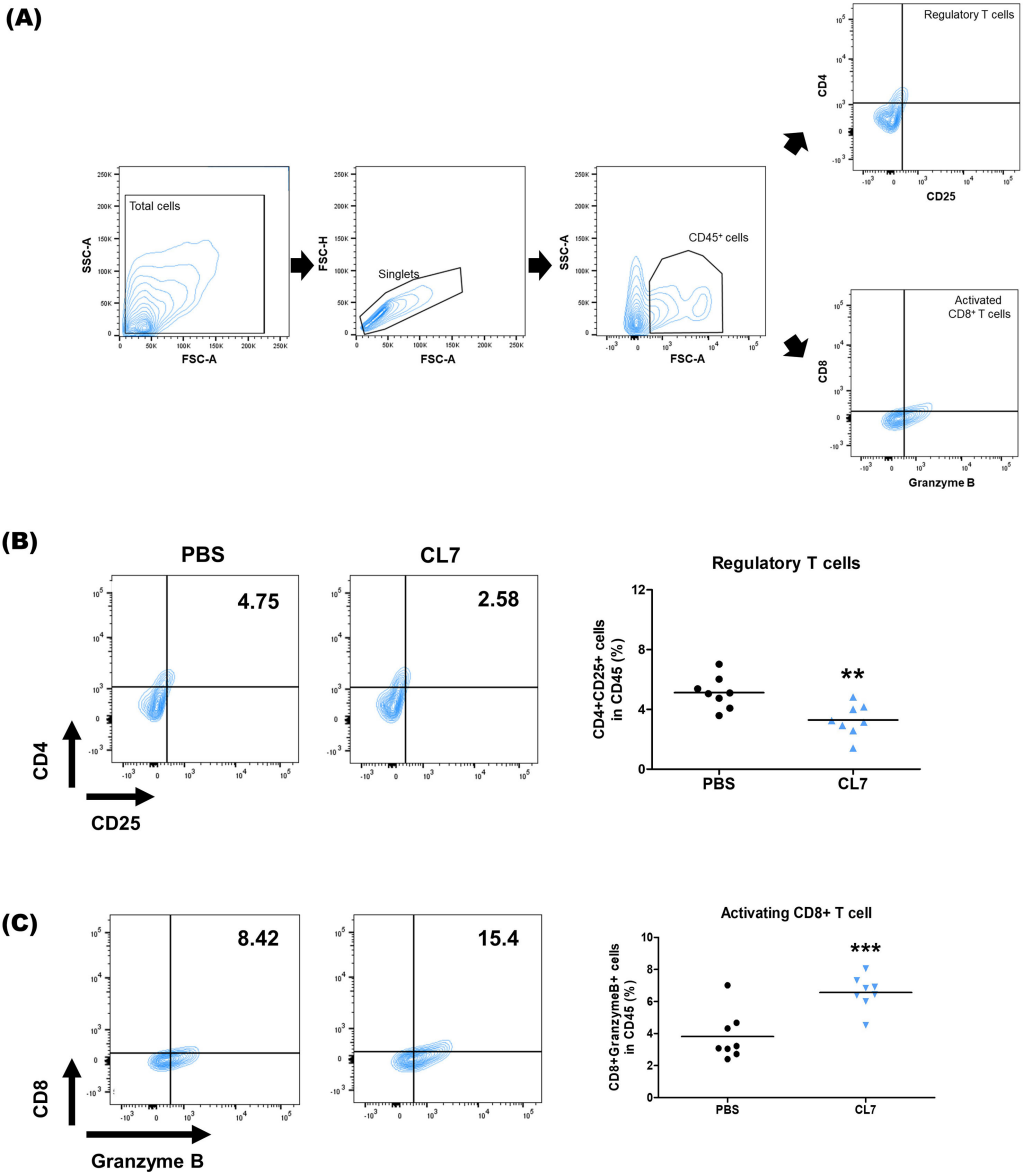
CL7 alleviates immunosuppressive conditions in the NSCLC TME. Identification of changes in immune cell populations within the NSCLC orthogonal mouse model by flow cytometry. Gating boundaries were defined based on unstained and single-stained controls used for compensation and negative reference. **(A)** Identification of lymphoid cell populations following exclusion of doublets. CD45^+^ gate was also used as a first step for specific immune cell identification. **(B)** CD45^+^CD4^+^CD25^+^ cells were considered infiltrating regulatory T cells (Tregs), and **(C)** CD45^+^CD8^+^GranzymeB^+^ cells were considered infiltrating activated CD8^+^ T cells. All data are presented as the mean ± SEM; ** *p* < 0.01 versus PBS group (n = 8 per group).

To investigate the effect of CL7 treatment on lymphoid cell populations, flow cytometry was performed on lung tissues from NSCLC-bearing mice (**Figure 4A**). A significant reduction in regulatory T cells (Tregs) was observed in the CL7-treated group compared to the PBS control (**Figure 4B**). Furthermore, the CL7 treatment significantly increased the CD8^+^ T cell populations (**Figure 4C**). These results suggest that CL7 may contribute to relieving the immunosuppression in the TME, accompanied by cytotoxic CD8^+^ T cells.

### 3.5 CL7 attenuates immunosuppression and enhances immune activation in NSCLC model

**Figure 5:**
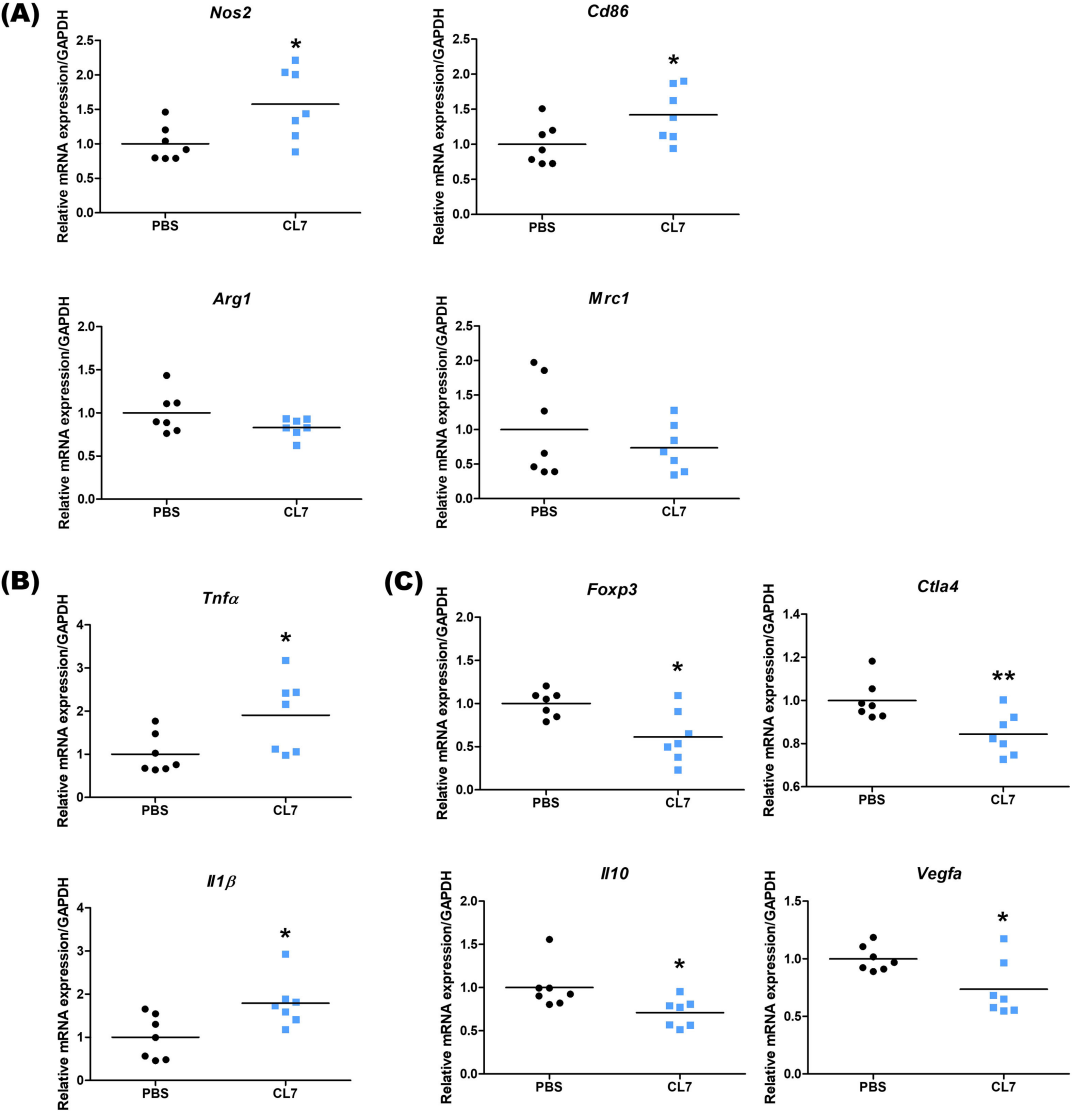
CL7 attenuates immunosuppression and enhances immune activation in NSCLC model. After the *in vivo* experiment, lung tissues from NSCLC orthotopic mouse model were harvested (n = 7 per group), and total RNA was extracted for qRT-PCR analysis. The mRNA expression levels of **(A)** Nos2 and Cd86 (M1 macrophage-related genes), Arg1 and Mrc1 (M2 macrophage-related genes), **(B)** Tnfα and Il1β (inflammation-related genes), and **(C)** Foxp3, Ctla4, Il10, and Vegfa (immunosuppressive genes) were quantified. All data are presented as mean ± SEM; * *p* < 0.05, ** *p* < 0.01 versus PBS group.

To evaluate the immunomodulatory effects of CL7 treatment in the NSCLC tumor microenvironment, we analyzed the expression levels of immune-related genes using qRT-PCR. The expression of M1 macrophage markers, Nos2 and Cd86, was significantly increased in the CL7-treated group compared to the PBS group, indicating the promotion of pro-inflammatory macrophage activation. In contrast, the expression levels of M2 markers, such as Arg1 and Mrc1, showed no significant changes (**Figure 5A**). CL7 treatment also upregulated the expression of inflammatory cytokines Tnfα and Il1β (**Figure 5B**). Notably, the expression of immunosuppressive markers, including Foxp3, Ctla4, Il10, and Vegfa, was significantly decreased in the CL7-treated group (**Figure 5C**). These findings suggest that CL7 may alleviate immunosuppression within the NSCLC tumor microenvironment and promote antitumor immune responses.

## 4 Discussion

Our study highlights the therapeutic potential of tumor-associated macrophage (TAM) reprogramming with immunotherapy for the treatment of non-small cell lung carcinoma (NSCLC). The main finding of our research is that CL7, an anti-CD300c antibody, promotes M1 macrophage polarization in the TME while concurrently reducing Treg infiltration, resulting in significant tumor volume reduction. The potential clinical significance of our findings is that TAM reprogramming successfully transformed the immune-suppressive TME into a pro-inflammatory environment, leading to a therapeutic effect.

NSCLC is the most prevalent subtype of lung cancer and is characterized by relatively slow but progressive growth, eventually acquiring metastatic potential (22). Despite advances in standard treatment modalities, including surgery, chemotherapy, and radiotherapy, therapeutic efficacy remains unsatisfactory, especially in late-stage NSCLC (23). The prognosis remains poor, with less than 5% of patients surviving beyond stage IV. Early detection is also limited by the asymptomatic nature of early-stage disease (24). One of the central challenges in NSCLC treatment is its immune evasive capacity, largely attributed to the highly immunosuppressive nature of the TME. Through mechanisms such as immune editing and mimicry of normal cells, tumor cells can escape immune surveillance and suppress effective antitumor immunity (25).

In this context, the tumor microenvironment (TME) has gained attention as a key determinant of tumor development and therapeutic response. The TME comprises not only tumor cells but also a diverse array of stromal and immune components, including endothelial cells, fibroblasts, immune cells, and extracellular matrix elements (26, 27). These cellular and non-cellular interactions dynamically shape tumor initiation and progression. Tumor-associated macrophages (TAMs), which are the main components in TME, have complex functions in terms of their antitumor or protumor effects. TAMs are broadly classified into classically activated M1 macrophages that produce effector molecules such as reactive oxygen and nitrogen intermediates, and TNFα, to limit tumor growth, and alternately activated M2 macrophages that promote tumor growth and metastasis by secretion of matrix-degrading enzymes, angiogenic factors and immunosuppressive cytokines/chemokines (28). The balance of these macrophages determines the anti- or protumor effects of the macrophage population within the TME.

CD300 molecules regulate immune responses by interacting with lipid-based ligands, with CD300c acting as an activator and CD300a as an inhibitor. CD300c is expressed on T cells, NK cells, macrophages, and neutrophils (29). It has been reported that CD300c modulates the activation of immune cells (30, 31). Previous studies have reported that targeting CD300c with monoclonal antibodies can stimulate MAPK and NF-κB signaling, thereby inducing M1 polarization. The CL7 antibody, developed by Lee et al., has shown promising antitumor activity in the CT26 colon cancer model through this mechanism (21). Based on these findings, we hypothesized that CL7 could similarly modulate the immune landscape in NSCLC. Previous study demonstrated that CL7 specifically binds to exogenously expressed CD300c on 293T cells and induces M1 macrophage differentiation through activation of the MAPK and NF-κB pathways. *In vitro*, CL7 also upregulated PD-L1 expression on THP-1 cells, further supporting its role in modulating myeloid activation through CD300c engagement (21). Building upon these findings, the present study extends the investigation to *in vivo* tumor models, showing that CL7 treatment markedly suppresses tumor growth and enhances M1 macrophage enrichment within the tumor microenvironment. Although these results suggest that CL7 exerts its antitumor effect, the direct *in vivo* evidence confirming complete CD300c dependence remains limited. To address this limitation, future studies using CD300c knockout mice and blocking-antibody approaches are needed to further validate the target specificity of CL7 and exclude potential off-target effects. Moreover, additional studies are warranted to clarify how CL7-mediated CD300c engagement triggers MAPK and NF-κB signaling in tumor-associated macrophages and how these pathways subsequently shape T cell activation. Such analyses will be important to delineate the molecular framework underlying CL7-induced immune modulation.

The dose range used in this study was selected based on our previous preclinical work, where 10 mg/kg was used for comparison with the anti-PD-1 antibody and consistently showed measurable therapeutic efficacy (21). 5 mg/kg was included as a lower reference dose to evaluate whether comparable effects could be achieved. The absence of a clear difference between these two doses suggests that CL7’s activity may begin to plateau within this range. To more precisely define the dose–response relationship and identify the optimal therapeutic window, additional experiments encompassing a broader range of concentrations (0.1–10 mg/kg) will be conducted in future studies. These efforts will also help determine whether specific immunological changes, such as macrophage polarization dynamics, are dose-dependent. In addition, considering future clinical applications, further studies using humanized models and toxicity studies are needed.

CL7 treatment notably expanded the M1 macrophage population without significantly altering other myeloid subsets (**Figures 3**, **5A**), suggesting that M1 macrophage enrichment plays a key role in the observed tumor suppression. This result aligns with previous reports showing a positive correlation between M1 macrophage density in tumor islets and prolonged survival in NSCLC patients (32, 33). Interestingly, no significant change was observed in M2 macrophage levels between the PBS- and CL7-treated groups, implying that the therapeutic effect of CL7 is primarily driven by the activation and infiltration of M1 macrophages rather than modulation of the M2 compartment. The observed increase in M1 macrophages without a corresponding decrease in M2 cells suggests that CL7 treatment primarily promotes the recruitment and differentiation of circulating monocytes into M1 macrophages, rather than direct M2-to-M1 repolarization within the tumor microenvironment. CD300c engagement activates pro-inflammatory signaling cascades in myeloid cells, potentially enhancing monocyte trafficking and M1-type differentiation upon tumor infiltration. This interpretation is consistent with our flow cytometry data, which show elevated M1 populations but stable M2 levels, indicating that CL7 expands the M1 compartment through *de novo* recruitment and activation rather than conversion of existing M2 macrophages. Collectively, these results suggest that CL7 remodels the tumor immune landscape toward a more proinflammatory, antitumor phenotype. Although these findings support the hypothesis that CL7 modulates macrophage polarization, they do not directly demonstrate M2-to-M1 repolarization *in vivo*. To better define the direct polarizing effect of CL7 on NSCLC-associated macrophages, further investigations—including immunohistochemical validation of macrophage phenotypes and *in vitro* co-culture experiments—are needed.

Regulatory T cells (Tregs), a key immunosuppressive cell population within the TME, are known to impair antitumor immunity and are associated with poor prognosis in NSCLC (34). Recent research in various cancer types has shown that insufficient glucose supply and increased intracellular glycolysis in cancer cells can lead to the production of lactic acid and fatty acids, which enhance Treg proliferation (35, 36). Additionally, tumor-derived factors such as TGF-β, ATP, and indoleamine 2,3-dioxygenase (IDO) have been shown to reinforce the immunosuppressive function of Tregs within the TME (37–39). The macrophage receptor with collagenous structure (MARCO), found on tumor-associated macrophages (TAMs), has been shown to stimulate Treg proliferation and IL-10 production in NSCLC (40). During the protumor inflammatory phase of lung cancer, TGF-α stimulation can increase MHC-II expression on alveolar type II cells, triggering Treg expansion and facilitating the development of inflammation-driven lung adenocarcinoma (41). In our study, CL7 resulted in a significant reduction in Tregs (**Figure 4B**), accompanied by decreased expression of immunosuppressive genes such as Foxp3, Ctla4, Il10, and Vegfa, indicating a partial regulation of the immunosuppressive TME (**Figure 5C**).

CD8^+^ T cells mediate antitumor immunity by recognizing and killing tumor cells through MHC class I-restricted antigen presentation, as well as releasing cytotoxic molecules such as perforin and Granzyme B (42, 43). However, M2-like tumor-associated macrophages have been shown to impair T cell receptor (TCR) signaling, thereby suppressing the activation and cytotoxic function of CD8^+^ T cells (44). As expected, activated CD8^+^ T cell population enhanced significantly (**Figure 4C**), suggesting that CL7 could promote cytotoxic T cell functions. In parallel, CL7 treatment induced a notable increase in M1-like macrophages without significantly affecting the M2 macrophage population, and upregulated pro-inflammatory cytokines including TNF-α and IL-1β (**Figure 5B**). These results suggest that CL7 modulates the innate immune landscape by promoting a pro-inflammatory macrophage phenotype, which may contribute to antitumor immunity. These findings indicate that CL7 partially remodels the immunosuppressive TME to an immune-stimulatory environment. However, further mechanistic studies are needed to elucidate the pathways underlying CL7-mediated immune modulation.

Taken together, our findings demonstrate that CL7, a CD300c-targeting antibody, exerts potent antitumor effects in NSCLC by reshaping the tumor immune microenvironment. By promoting M1-like macrophage polarization, suppressing regulatory T cells, and enhancing cytotoxic T cell activity, CL7 shifts the TME from an immunosuppressive state toward a pro-inflammatory, tumor-inhibitory landscape. Further studies are needed to elucidate the underlying molecular mechanisms and assess their potential for clinical translation.

## Data Availability

The datasets presented in this study can be found in online repositories. The names of the repository/repositories and accession number(s) can be found in the article/**Supplementary Material**.
