# Supplementary material for: The CD300c antibody CL7 suppresses tumor growth by regulating the tumor microenvironment in non-small cell lung carcinoma
Source: Front Oncol. 2025 Nov 25;15:1698857. doi: 10.3389/fonc.2025.1698857 (PMC12685643; doi:10.3389/fonc.2025.1698857)
Supplement: Supplementary file 1 [file Table1.docx]

Supplementary Table 1. qRT-PCR primers for analysis

| **Gene** | **F/R** | **Primer Sequence** |
| --- | --- | --- |
| **GAPDH** | F | 5’-ACC CAG AAG ACT GTG GAT GG-3’ |
|  | R | 5’-CAC ATT GGG GGT AGG AAC AC-3’ |
| **Mrc1** | F | 5’-TTC GGT GGA CTG TGG ACG AGC-3’ |
|  | R | 5’-ATA AGC CAC CTG CCA CTC CGG-3’ |
| **Arg1** | F | 5’- AGA CAG CAG AGG AGG TGA AGA G-3’ |
|  | R | 5’-CGA AGC AAG CCA AGG TTA AAG C-3’ |
| **Cd86** | F | 5’-GAC CGT TGT GTG TGT TCT GG-3’ |
|  | R | 5’-GAT GAG CAG CAT CAC AAG GA-3’ |
| **Nos2** | F | 5’-GGC AGC CTG TGA GAC CTT TG-3’ |
|  | R | 5’-GAA GCG TTT CGG GAT CTG AA-3’ |
| **Tnfα** | F | 5’-CAT CTT CTC AAA ATT CGA GTG ACA A-3’ |
|  | R | 5’-TGG GAG TAG ACA AGG TAC AAC CC-3’ |
| **Il1β** | F | 5’-GCC CAT CCT CTG TGA CTC AT-3’ |
|  | R | 5’-AGG CCA CAG GTA TTT TGT CG-3’ |
| **Foxp3** | F | 5’-ACC ATT GGT TTA CTC GCA TGT-3’ |
|  | R | 5’-TCC ACT CGC ACA AAG CAC TT-3’ |
| **Ctla4** | F | 5’-AGA ACC ATG CCC GGA TTC TG-3’ |
|  | R | 5’-CAT CTT GCT CAA AGA AAC AGC AG-3’ |
| **Il10** | F | 5’-CCA AGC CTT ATC GGA AAT GA-3’ |
|  | R | 5’-TTT TCA CAG GGG AGA AAT CG-3’ |
| **Vegfa** | F | 5’-CCA CGA CAG AAG GAG AGC AGA AGT CC-3’ |
|  | R | 5’-AGG TAA CGC CAG GAA TTG TTG C-3’ |

**Supplementary Table 1. Primers used in Real-Time PCR.**
